# A regional consensus recommendation on brain atrophy as an outcome measure in multiple sclerosis

**DOI:** 10.1186/s12883-016-0762-5

**Published:** 2016-11-24

**Authors:** Raed Alroughani, Dirk Deleu, Khalid El Salem, Jasem Al-Hashel, K. John Alexander, Mohamed Assem Abdelrazek, Adel Aljishi, Jaber Alkhaboori, Faisal Al Azri, Nahida Al Zadjali, Majed Hbahbih, Tag Eldin Sokrab, Mohamed Said, Àlex Rovira

**Affiliations:** 1 Division of Neurology, Department of Medicine, Amiri Hospital, Kuwait City, Kuwait; 2 Neurology Clinic, Dasman Diabetes Institute, Dasman, Kuwait; 3 Division of Neurology (Neuroscience Institute), Hamad General Hospital, Doha, Qatar; 4 Department of Neurology, Jordan University of Science and Technology, King Abdullah University Hospital, Irbid, Jordan; 5 Department of Neurology, Ibn Sina Hospital, Kuwait City, Kuwait; 6 Department of Radiology, Ibn Sina Hospital, Kuwait City, Kuwait; 7 Department of Neurology, Salmaniya Hospital & AGU, Manama, Bahrain; 8 Department of Neurology, Royal Hospital, Muscat, Oman; 9 Department of Radiology, Sultan Qaboos University Hospital, Muscat, Oman; 10 Department of Radiology, Royal Hospital, Muscat, Oman; 11 Royal Medical Services, Amman, Jordan; 12 Division of Neurology (Neuroscience Institute), Hamad General Center, Doha, Qatar; 13 Medical Manger-Gulf Countries, Novartis pharmaceuticals, Dubai, United Arab Emirates; 14 Department of Radiology, Hospital Universitari Vall d’Hebron, Barcelona, Spain

**Keywords:** Multiple sclerosis, Brain atrophy, Consensus, NEDA, Cognitive impairment, Disability progression, Middle East

## Abstract

**Background:**

Multiple sclerosis (MS) is a chronic autoimmune disease characterized by inflammatory and neurodegenerative processes leading to irreversible neurological impairment. Brain atrophy occurs early in the course of the disease at a rate greater than the general population. Brain volume loss (BVL) is associated with disability progression and cognitive impairment in patients with MS; hence its value as a potential target in monitoring and treating MS is discussed.

**Methods:**

A group of MS neurologists and neuro-radiologists reviewed the current literature on brain atrophy and discussed the challenges in assessing and implementing brain atrophy measurements in clinical practice. The panel used a voting system to reach a consensus and the votes were counted for the proposed set of questions for cognitive and brain atrophy assessments.

**Results:**

The panel of experts was able to identify recent studies, which demonstrated the correlation between BVL and future worsening of disability and cognition. The current evidence revealed that reduction of BVL could be achieved with different disease-modifying therapies (DMTs). BVL provided a better treatment and monitoring strategy when it is combined to the composite measures of “no evidence of disease activity” (NEDA). The panel recommended a set of cognitive assessment tools and MRI methods and software applications that may help in capturing and measuring the underlying MS pathology with high degree of specificity.

**Conclusion:**

BVL was considered to be a useful measurement to longitudinally assess disease progression and cognitive function in patients with MS. Brain atrophy measurement was recommended to be incorporated into the concept of NEDA. Consequently, a consensus recommendation was reached in anticipation for implementation of the use of cognitive assessment and brain atrophy measurements on a regional level.

## Background

MS affects over 2.5 million people worldwide, mainly female adults [1]. In the Middle East and North Africa region, the overall MS prevalence was reported to be 51.52/100,000 [2]. MS is characterized by inflammation and neurodegeneration resulting in irreversible neurological impairment [3, 4]. The acute inflammation component, which occurs during the early phases of the disease, is responsible for the relapses whereas the progressive phase is characterized by the axonal damage leading to accumulation of disability. In addition, brain atrophy occurs in all stages of MS, starting at early stages and progresses throughout the course of the disease at a higher rate than atrophy associated with the normal aging process of healthy individuals [5]. Several disease modifying therapies (DMTs) have shown better efficacy in reducing the clinical and radiological activities which may potentially halt the accumulation of axonal damage and consequently reduce the rate of BVL [6–8]. Recent studies have prompted debates about the clinical relevance of BVL over time as a measure to quantify neurodegeneration. Measuring brain atrophy through magnetic resonance imaging (MRI) new techniques and software applications development allows better and reliable assessments of brain volume, monitors changes longitudinally, and assesses treatment effects that could be implemented in the routine clinical practice [9].

## Methods

A group of thirteen neurologists and neuro-radiologists with expertise in MS from Bahrain, Jordan, Kuwait, Oman, Qatar, and Spain met to address the unmet needs of assessing brain atrophy in patients with MS, discuss the relevant parts of the brain in terms of disease progression with MS. A comprehensive literature search was performed of MEDLINE, EMBASE, and Cochrane databases, systematically reviewing all manuscripts between January 1st, 1995 and May 10th, 2016. The panel identified the relevant literature, using the following MeSH terms: ‘Disease Modifying therapies’ AND ‘multiple sclerosis’ AND ‘brain atrophy’. Additional searches were performed of American Academy of Neurology (AAN) and European Committee for Treatment and Research in Multiple Sclerosis (ECTRIMS) abstracts for the last two years, using identical search strategies on their respective websites. The recent updates and emerging data in measuring BVL and its clinical relevance were discussed. Different MRI-based methods, protocols, and the challenges in applying them into clinical practice were reviewed with emphasis on the emerging data of immediate and long-term effects of DMTs on brain atrophy. A voting system was followed by the panel to reach a consensus and the votes of the experts were counted for the proposed set of questions for each assessment. The consensus was based on the highest number of votes for each single question of each assessment. Finally, a consensus recommendation on how brain volume and cognition need to be assessed and implemented in clinical practice on a regional level was developed.

## Results

### Implications of brain volume loss in MS and unmet needs

MS causes focal and diffuse damage to the brain. The focal white matter lesions are the classic hallmark of MS and appear on MRI as T_2_, gadolinium enhanced T_1_ lesions, or T_1_-hypointense lesions (black holes) [10]. The damage also occurs in grey matter as well as diffusely in normal appearing white matter and consequently lead to BVL [11, 12]. Among MRI measures, BVL in MS is considered as one of the prognostic measures which has been directly correlated with cognitive impairment [13, 14], disability progression [15–17], and fatigue [18]. A number of longitudinal studies showed that BVL predicts future worsening of disability and cognition. A 10-year follow-up study demonstrated that brain atrophy occurred throughout the course of the disease and was more severe in the group that showed disability progression at 5 years of follow-up. Furthermore, it was demonstrated that the overall grey matter atrophy was a better predictor of disease progression than white matter atrophy during the same follow-up period [19]. In a 13-year follow-up of 75 MS patients, Fillipi et al. showed that grey matter damage was associated with significant worsening of disability and cognitive function in 66% and 34% of patients, respectively [20]. In a study of 261 MS patients, brain atrophy and lesion load were complimentary predictors of long term disability over a 10-year follow-up period [21]. BVL has been observed at the early phases of the disease in patients with clinically isolated syndrome (CIS) [5].

With respect to age, it has been largely known that after adolescence, grey matter volume starts to decline in a linear way (−0.09%/year) while white matter decline starts after midlife, and at a higher rate (−0.2%/year) [22, 23]. Regarding cortical thickness, at midlife a global thinning was apparent, spreading over several cortical regions [24]. The decline in the thickness measured was around 0.016 mm/decade. Both volumetric and cortical thickness age-associated changes had been described, implying that age is a variable that should be considered when assessing global or regional brain volume in MS patients.

There were few reported studies exploring potential gender-associated differences and brain size. Men had larger grey and white matter volumes compared to women although after adjusting by total intracranial volume, these differences disappeared [22]. Regarding cortical thickness, men showed slightly larger cortical thickness compared to females only at midlife, and the rate of progressive thinning was similar for both groups [24]. A recent study reported that cortical thickness was associated with total intracranial volume, but not with gender [25]. Since gender and total intracranial volume are highly correlated, it is a matter of debate whether one or both variables should be considered when assessing global or regional brain volume in MS patients [26, 27].

The participants also identified several unmet needs regarding brain volume measurements and its implementation in the routine clinical practice (Table 1).

**Table 1:** A list of unmet needs with brain volume as identified by the panel

Unmet Needs	
1.	Applicability of MRI volume measurement in clinical practice
2.	Lack of large long-term prospective studies of clinical correlates with brain volume loss
3.	Reliable assessments of cognitive impairment in MS
4.	Validation of disease progression parameters with brain volume loss
5.	Lack of pathological correlation with brain volume loss
6.	Paucity of data on regional brain volume effect in MS
7.	Targeting brain volume loss as one of the main outcome measure in clinical trials
8.	Reproducible effectiveness of DMTs in phase III studies on cognitive impairment

### Assessment of brain volume in patients with MS

Conventional MRI techniques are considered the gold standard for diagnosing and monitoring the response to treatment in patients with MS. However, conventional MRI has limited specificity and correlation with disability measures [28, 29]. In addition, conventional MRI does not offer any information about the ongoing degenerative/reparative processes. Therefore, the panel discussed the current MRI methods and software applications that help in capturing and measuring the underlying MS pathology with high degree of specificity. Currently, segmentation- and registration-MRI based methods are used to measure brain volume. The segmentation-based method such as brain parenchymal fraction (BPF), white matter fraction (WMf), grey matter fraction (GMf), and normalized brain volume (NBV) provide assessment of global (BPF, NBV) or regional (WMf, GMf) brain volume at a single time-point in an automated way [28–31]. However, the data extracted out of this method is usually heterogeneous and influenced by the quality of T1-weigthed images acquired, and are not recommended for longitudinal analysis. Registration-based methods measure brain volume at two time points, in order to calculate the percentage brain volume change (PBVC). These methods are robust, sensitive to changes over time, less influenced by the disruption of the quality of MR imaging acquisitions, and are most suitable for evaluating global brain volume changes but are not usually designed to analyse regional volume changes over time [32]. The sensitivity and the specificity could vary according to the cut-off value that identify the annualized PBVC. With a cut-off value of 0.4%, the sensitivity and specificity are 65% and 80%, respectively [32]. The panel listed the advantages and disadvantages for brain volume assessment in patients with MS (see Table 2.)

**Table 2:** Advantages and disadvantages of brain volume assessment in MS

Advantages	
	Whole brain atrophy is easy to measure
	Brain atrophy is a “summary measure” of the irreversible/destructive pathological process of MS
	Whole brain atrophy is highly reproducible and sensitive to disease-related changes
	Correlates with disability, cognitive impairment and fatigue
Disadvantages:	
	End-stage phenomenon
	Pseudoatrophy effect (first 6–12 months)
	Fluctuations: steroids, hydration
	Co-morbidities: smoking, alcohol, high BMI, etc.
	MRI technical confounding factors
	Time consuming: reimbursement?
	Not enough evidence to use atrophy measures to assess and predict individual treatment response

*BMI* Body Mass Index

### Disease-modifying therapies effect on brain volume

It is important to target the neurodegenerative process in MS patients, in addition to the inflammation component. In general, the results of brain volume data vary from one study to another, which could be attributed to the different mechanism of action of DMTs, the ability of these drugs to cross the blood brain barrier and the heterogeneity of the patient population. In phase III clinical trials, several DMTs demonstrated reduction in the rate of brain volume loss in relapsing-remitting multiple sclerosis (RRMS) patients at variable levels with different response time (Table 3.) [6–8, 33–49]. However, one should be careful in interpreting data across trials with different patient population, using different methods in measuring brain volume. In both placebo-controlled as well as active comparator trials, most of the DMTs provided a delayed effect around the second year of treatment with the exception of daclizumab and fingolimod [6–8, 33–54]. In the FREEDOMS trials, fingolimod demonstrated an effect on brain volume within 6 months of treatment [6, 8]. The first reported brain atrophy study with intramuscular (IM) interferon (IFN)-β-1a demonstrated lower rate of BVL than placebo in the second year of treatment of RRMS patients (−0.23% IFN-β-1a compared to −0.51% in the placebo group; *p* = 0.03) [33]. However, the subcutaneous (sc) IFN-β-1a produced conflicting results in both CIS and RRMS patients [33, 34, 50, 51]. Data from placebo controlled trials on sc IFN-β-1b and BVL in RRMS patients are not published to date [52, 53]. Glatiramer significantly reduced BVL by 40% at 9–18 months and 25% at 0–18 months compared to placebo [35]. Three trials compared glatiramer and IFN-β and showed a marginal reduction in BVL in one study [36–38]. In two trials, natalizumab increased the rate of BVL in the first year and then significantly reduced it versus placebo in the second year [40, 41]. Teriflunomide failed to demonstrate reduction in the rate of BVL compared to placebo [42]. Dimethyl fumarate produced a 21% reduction in BVL compared to placebo in the DEFINE study and failed to show any statistically significant reduction in BVL in the CONFIRM study [43, 44]. In the CARE-MS I and II, alemtuzumab reduced the rate of BVL to 24–42% compared to sc IFN-β-1a [45, 46]. Both the ALLEGRO and the BRAVO studies reported reduction of brain atrophy with laquinimod [47, 48]. Daclizumab had a marginal, but a significant favorable effect on brain atrophy compared to IM IFN-β-1a [49]. In the three trials (FREEDOMS I & II and TRANSFORMS), fingolimod also significantly reduced BVL to 28–45% and the reduction of brain atrophy was evident early during the course of the treatment [6–8].

**Table 3:** The effect of DMTs on BVL in RRMS patients in Phase III trials

Drug (REF.)	Changes in Brain Volume Loss		
	Year 0–1	Year 1–2	Year 0–2
IFN-β-1a IM [33]	x	✓ 55% reduction vs. placebo	x
IFN-β-1a SC [34]	-	-	x
IFN-β-1b SC [52, 53]	-	-	-
Glatiramer acetate [35, 36–38]	x	✓	x
	(Eur/Canadian GA trial)	40% reduction vs. placebo (Eur/Canadian GA trial)	(Eur/Canadian GA trial)
	8% reduction vs. sc IFN-β-1a (REGARD)^+^	22% reduction vs. sc IFN-β-1a (REGARD)^+^	13% reduction vs. IFN-β-1a (REGARD)
	No sig. difference with GA +/− sc IFN-β-1b (BEYOND)	No sig. difference with GA +/−. sc IFN-β-1b (BEYOND)	No sig. difference with GA +/− sc IFN-β-1b (BEYOND
	No sig. difference with GA +/− sc IFN-β-1a (COMBIRx)	No sig. difference with GA +/−. sc IFN-β-1a (COMBIRx)	No sig. difference with GA +/− sc IFN-β-1a (COMBIRx)
Natalizumab [40, 41]		✓	x (AFFIRM)
	40% increase vs. placebo (AFFIRM)	44% reduction vs. placebo (AFFIRM)	
	19% increase vs. placebo (SENTINEL)	x 23% reduction with Natalizumab+ IM IFN-β-1a vs. IM IFN-β-1a + placebo (SENTINEL)	x (SENTINEL)
Teriflunomide [42]	37% reduction vs. placebo (TEMSO)	31% reduction vs. placebo (TEMSO)-	X
Dimethyl fumarate [43, 44]	-	21% reduction vs. placebo (DEFINE) Significant effect (DEFINE) ×‡ (CONFIRM)	✓21% reduction vs. placebo (DEFINE) × ‡ ()
Alemtuzumab [45, 46]		-	✓ 24–42% reduction vs IFN-β-1a
Laquinimod [47, 48]	-	-	✓ (ALLEGRO) 33% reduction vs placebo (BRAVO) 28–34% reduction vs placebo
Daclizumab [49]	✓	✓	-
	Significant effect (Week 0–24) 9% reduction vs IM IFN-β-1a	Significant effect (Week 24–96) 7% reduction vs IM IFN-β-1a	
Fingolimod [6–8]	✓	✓	✓
	23–40% reduction vs placebo	28–45% reduction vs placebo	33–35% reduction vs placebo
	✓§ 45% reduction vs. IM IFN β-1a (TRANSFORMS)	-	-

*BID* twice daily, *TID* three times daily, SC subcutaneous, *GA* Glatiramer acetate, *IFN* Interferon

– Data not reported/available × No significant effect or not statistically significant ✓ Significant effect

* Not all approved therapies have significant effects on BVL and effects can be delayed until the second year of therapy. + No *P* value reported †Significant effect at 9–18 months

‡Significant effect at 6–24 months in DEFINE (only BID, not TID dose arm), but not in CONFIRM study

§Significant effect also seen at 0–6 months

### New emerging strategies in MS

**Fig. 1:**
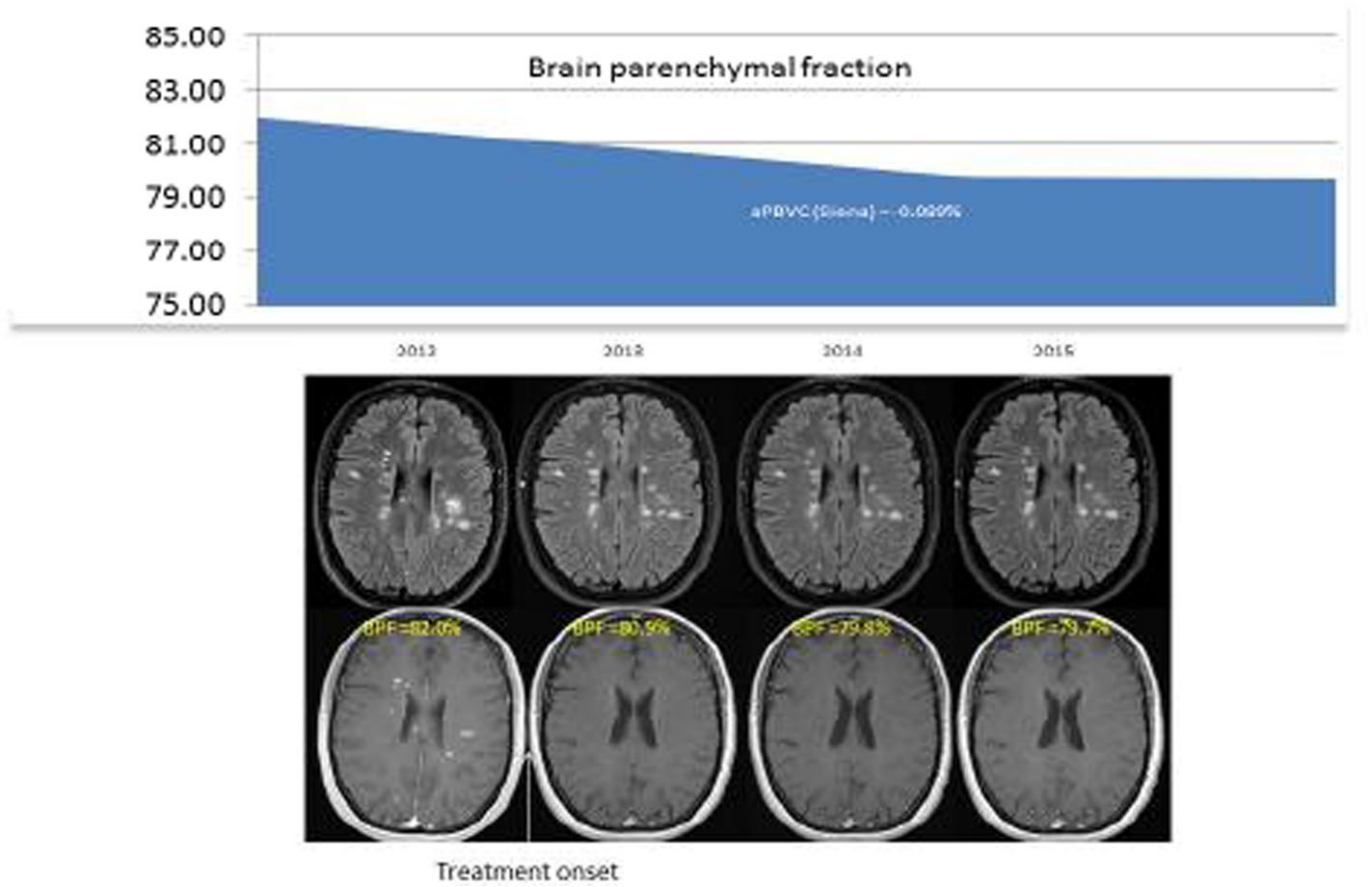
Illustration of a longitudinal effect of treatment on brain parenchymal fraction. (Source: Dr. Rovira; data on file)

The panel discussed incorporating BVL as a key measure and part of the treatment strategy, which focuses on achieving no evidence of disease activity (NEDA). The measures under NEDA-3 were focused on the composite measures of absence of relapses, disability progression (based on expanded disability status scale (EDSS) scores), and MRI activity (new or enlarging of T_2_ lesion) [30, 31]. As more evidence is accumulating with respect to the importance of brain atrophy during different stages of MS, absence of BVL may be incorporated to the NEDA measures to be a total of four measures (NEDA-4). Incorporating BVL in NEDA-4, allows a more comprehensive and balanced assessment, capturing both focal and diffuse disease activity [55, 56]. In the pooled analysis of the two FREEDOMS studies with fingolimod, patients on fingolimod were 4 times were more likely to achieve NEDA-4 than those who were on placebo at two years [57]. The advisors discussed the following case as an example of a patient who achieved a NEDA-4 during a period of 3 years. A 35-year old female was diagnosed with MS and had a highly active disease (more than 20 active lesions) at baseline. A disease modifying therapy was initiated in 2012. Over the 3-year longitudinal follow-up, there was no evidence of disease activity (absence of relapses, disease progression and MRI new/enlarging lesions). The change in the annualized brain volume change during the observational period was −0.089, which was below the threshold considered in NEDA 4 of −0.4% (Fig. 1).

### Cognitive assessment consensus

The panel acknowledged that cognitive ability is not readily observable in routine neurological examinations, and self-reported cognitive complaints are confounded by mood and other subjective symptoms. One of the core cognitive deficits in MS patients is slowing of information processing speed, which can be subtle and difficult to assess without formal neuropsychological testing. In addition, there is a problem with the testing-retesting reliability as well as confounders at the time of testing. The advisors listed the most common tools that are utilized for cognitive assessment in their clinical practice such as the BICAMS (Brief International Assessment for Cognition for MS), CDT (The Clock Drawing Test), the MSNQ (MS Neuropsychological Screening Questionnaire), the PASAT (The Paced Auditory Serial Addition Task), the SDMT (Symbol Digit Modalities Test), and finally the WLG (Word List Generation) (Table 4.).

**Table 4:** The tally of cognition assessment

How?	SDMT	BICAMS	PASAT	WLG	CDT
	9	3	4	1	4
In Whom?	RIS	CIS	RRMS	SPMS	
	7	8	12	8	
When?	Initial	3–6 months	12 months		
	2	1	12		
Where?	Office	Home	Waiting area		
	12	1	0		
Who?	Physician	Nurse	Assistant	Automated	
	1	10	4	3	

*Abbreviations*: *BICAMS* (Brief International Cognitive Assessment for MS), *CDT* (The Clock Drawing Test), *PASAT* (The Paced Auditory Serial Addition Task), Symbol Digit Modalities Test (*SDMT*), *WLG* (Word List Generation)

The panel utilized a standard format of questions for assessing cognition, including; 1) the types of tools they would use to test cognitive assessment, 2) the types of MS patients in whom they would apply these tests 3) the timing of the assessment 4) where they would apply these tests 5) and finally who would apply these tools. They used a voting system to rate and highlight the importance and the clinical relevance of each cognition assessment test the in the real world setting. Each voting participant could vote multiple times for different categories.

Of the 12 expert voting panel, 75% voted for the Symbol Digit Modalities Test (SDMT) as the most common test they would utilize for cognition. All voted to use the SDMT in RRMS and equal distribution of votes for the rest of the MS spectrum. All of the advisors recommended to have the assessment test at 12 months during the office visit and 83% of the experts recommended the tools to be conducted by the nurse, since it is time consuming for most neurologists (Table 4).

### Brain volume assessment consensus

The advisors utilized the same methodology for assessing brain volume, including; 1) the types of methods they would use to assess BVL, 2) the types of MS patients they would target 3) the timing of the assessment during disease activity 4) and who would conduct these studies. They also used a voting system to rate importance and the clinical applicability of these methods in the routine clinical practice and each voting member was able to vote multiple times for different categories.

As for the brain volume assessment (Table 5.), all (100%) of the advisors recommended the Structural Image Evaluation, using Normalization, of Atrophy (SIENA), a registration-based method, to measure BVL mainly in patients with radiologically isolated syndrome (RIS), CIS, & RRMS at the 6–12 month time frame for most participants. It was recommended to have this measurement taken by the MRI technician.

**Table 5:** The tally of brain volume assessment

How?	SIENA	SIENAx	BPF	VBM	
	12	4			
In Whom?	RIS	CIS	RRMS	SPMS	
	7	11	12	4	
When?	Initial	3–6 months	Anytime with MRI acquisition	6–12 months	Annual
	3		2	11	1
Who?	Neurologist	Radiologist	MRI Technician	Independent	
		1	12	1	

*Abbreviations*: *BPF* (Brain Parenchymal Fraction), *SIENA* (Structural Image Evaluation, using Normalization, of Atrophy), *SIENAx* (Structural Image Evaluation, using Normalization, of Atrophy Cross-sectional), *VBM* (Voxel-Based Morphometry)

## Discussion

The expert panel considered BVL a relevant measure of diffuse damage and global marker for neuronal loss and also considered BVL to be a useful measurement to follow up patients with MS. The panel acknowledged that there are several confounders that should be taken into consideration before interpreting the results of these methods. The challenges include issues such non-standardization, variability, high technical expertise and infrastructure requirements, the availability of resources to perform the post-processing comparisons of the images, cost and reimbursements. In addition, there are MRI-related factors, for example variation in imaging protocols, artifacts such as signal heterogeneity, spatial distortions, motion, and the lack of normative data acquired with the same MR protocol. Other confounding factors include pseudoatrophy effect (BVL secondary to reduction in inflammation due to DMTs), and other non-MS related factors such as high body mass index, genetic factors, high alcohol consumption, smoking, dehydration, and cardiovascular risk factors [30, 31]. These challenges should be addressed before considering the implementation of these methods into clinical practice.

Currently, brain atrophy is not measured routinely in clinical practice to longitudinally assess the clinical progression and monitor treatment. Furthermore, brain atrophy measurement requires standardization of MRI acquisition and software techniques to allow proper comparisons at different time points during disease process. As mentioned previously, one should be aware of the DMT-induced pseudoatrophy effect, particularly, relevant in the first few months of treatment and it is more evident with highly anti-inflammatory DMTs. This phenomena is mainly seen in the white matter [30, 31].

The panel also recognized the importance of BVL as a cornerstone of measurement in NEDA-4 in the clinical progression and monitoring of MS, which should lead to improvement in treatment strategies and patient outcomes. As a result, the advisors recommended conducting further validation of NEDA-4 on a regional level to determine whether patients who achieve the four measures in NEDA-4 are at lower risk of future disability than those with disease activity or those achieving NEDA-3.

The panel agreed on the need for a consensus recommendation to share knowledge and opinions among experts in the field in order to unify aspects of clinical and radiological assessments. In addition, the advisors’ aspiration to deliver stronger messages to local neurologists may lead to ultimately improve the overall care of patients. The rationale for the consensus recommendations is not to focus on the inflammatory component but rather, to understand the neurodegenerative component of the disease process and to implement strategies to assess cognitive functions and brain atrophy over time. Studying the evolution and longitudinal changes of cognition and brain volume over time may shed light on different aspects of disease progression and the overall natural history of disease. The current diagnostic criteria lack any relevance to the neurodegenerative aspects and concentrate mainly on the inflammatory process. Indeed, the panel acknowledged that cognition and brain volume assessments might contribute to the overall treatment strategy.

## Conclusion

BVL occurs early and continues throughout the course of MS. It is considered to be a valid outcome measure to assess brain tissue loss and one of the best prognostic parameters of disability progression over the long term in patients with MS. BVL is accelerated in MS and correlates with disability and cognitive decline. Incorporating BVL to the composite measures of NEDA-3 is a shift in treatment and monitoring strategy to ultimately improve patient outcome measures. Advanced imaging and processing techniques will enable neurologists to probe diffuse changes in MS along with the clinical endpoints to monitor changes and ultimately improve treatment. Considerations should be made prior to system wide implementation of BVL measurement in clinical practice. 

## Abbreviations

AAN: American Academy of Neurology
AFFIRM: Natalizumab Safety and Efficacy in Relapsing-Remitting Multiple Sclerosis
ALLEGRO: Assessment of Oral Laquinimod in Preventing Progression in Multiple Sclerosis
BEYOND: Betaferon Efficacy Yielding Outcomes of a New Dose
BICAMS: Brief International Assessment for Cognition for MS
BID: twice daily
BMI: Body Mass Index
BPF: Brain Parenchymal Fraction
BRAVO: Benefit-Risk Assessment of Avonex and Laquinimod
BVL: Brain Volume Loss
CDT: Clock Drawing Test
CIS: Clinically Isolated Syndrome
CombiRx: Combination Therapy in Patients With Relapsing-Remitting Multiple Sclerosis
CONFIRM: Comparator and an Oral Fumarate in Relapsing–Remitting Multiple Sclerosis
DEFINE: Determination of the Efficacy and Safety of Oral Fumarate in Relapsing–Remitting Multiple Sclerosis
DMTs: Disease Modifying Therapies
ECTRIMS: European Committee for Treatment and Research in Multiple Sclerosis
EDSS: Expanded Disability Status Scale
FREEDOMS: FTY720 Research Evaluating Effects of Daily Oral therapy in Multiple Sclerosis
GA: Glatiramer acetate
GMf: Grey Matter fraction
IFN: Interferon
IM: Intramuscular
MeSH: Medical Subject Headings
MRI: Magnetic Resonance Imaging
MS: Multiple Sclerosis
MSNQ: MS Neuropsychological Screening Questionnaire
NBV: Normalized Brain Volume
NEDA: No Evidence of Disease Activity
PASAT: Paced Auditory Serial Addition Task
PBVC: Percentage Brain Volume Change
REGARD: the REbif vs Glatiramer Acetate in Relapsing MS Disease
RRMS: Relapsing-Remitting Multiple Sclerosis
SC: Subcutaneous
SDMT: Symbol Digit Modalities Test
SENTINEL: The Safety and Efficacy of Natalizumab in Combination with Interferon Beta-1a in Patients with Relapsing Remitting Multiple Sclerosis
SIENA: Structural Image Evaluation, using Normalization, of Atrophy
SIENAx: Structural Image Evaluation, using Normalization, of Atrophy Cross-sectional
TEMSO: Teriflunomide Multiple Sclerosis Oral
TID: Three times daily
TRANSFORMS: Trial Assessing Injectable Interferon versus FTY720 Oral in Relapsing-Remitting Multiple Sclerosis
VBM: Voxel-Based Morphometry.
WLG: Word List Generation
WMf: White Matter fraction
